# Supplementary material for: Heat adaptation of phage T7 under an extended genetic code
Source: Virus Evol. 2021 Dec 1;7(2):veab100. doi: 10.1093/ve/veab100 (PMC8923235; doi:10.1093/ve/veab100)
Supplement: veab100_Supp [file veab100_supp.zip › Sequencing_Analysis.zip!/Sequencing_Analysis/S2/evidence/SNP_6.html]

BRESEQ :: Evidence


| Predicted mutation | | | | | | |
| --- | --- | --- | --- | --- | --- | --- |
| evidence | seq id | position | mutation | annotation | gene | description |
| RA | NC\_001604.1 | 21,265 | A→C | intergenic (–/–) | – / – | –/– |

| Read alignment evidence... | | | | | | | | | | | |
| --- | --- | --- | --- | --- | --- | --- | --- | --- | --- | --- | --- |
|  | seq id | position | | ref | new | freq | score (cons/poly) | reads | annotation | genes | product |
| \* | NC\_001604.1 | 21,265 | 0 | A | C | 99.6% | 16435.1 / NA | 5282 | intergenic (–/–) | *–/–* | –/– |
| Reads supporting (aligned to +/- strand):  **ref** base A (11/11);  **new** base C (2550/2694);  **total** (2573/2709) | | | | | | | | | | | |
| Fisher's exact test for biased strand distribution *p*-value = 1.00e+00 | | | | | | | | | | | |
| Kolmogorov-Smirnov test that lower quality scores support variant *p*-value = 1.00e+00 | | | | | | | | | | | |

Only 100 of 5287 total aligned reads displayed.  

`GACTTATCCTAAAGAGGCTTGCCCATACATCCCGATTCGGATGGTCAGACTAGATGGTGAATCCTACGGTCGTTCGTACATTGAGGAATACTTAGGTGACTTACGGTCCCTTGAAAATCTCCAAGAGGCTATCGTCAAGATGTCCATGATTAGCTCTAAGGTTATCGGCTTAGTGAATCCTGCTGGTATCACCCAGCCACGCCGACTGACCAAAGCTCAGACTGGTGACTTCGTTACTGGTCGTCCAGAAGACATCTCGTTCCTCCAACTGGAGAAGCAAGCAGACTTTACTGTAGCTAAAGCCGTAAGTGACGCTATCGAGGCTCGCCTTTCGTTTGCCTTTATGTTGAACTCTGCGGTTCAGCGTACAGGTGAACGTGTGACCGCCGAAGAGATTCGGTATGTAGCTTCTGAACTTGAAGATACTTTAGGTGGTGTCTACTCTATCCTTTCTCAAGAATTACAATTGCCTCTGGTACGAGTGCTCTTGAAGCAACTACAAGCCACGCAACAGATTCCTGAGTTACCTAAGGAAGCCGTAGAGCCAACCATTAGTACAGGTCTGGAAGCAATTGGTCGAGGACAAGACCTTGATAAG  >  NC_001604.1/20965‑21562`  

                                                                                                                                                                                                                                                                                                            `|`                                                                                                                                                                                                                                                                                                           
  `gagacagtacagatGCTTGCCCATCCCTCCCGATTCGGATGGTCAGACTAGATGGTGAATCCTACTGTCGTTCGTACATTGAGGAATACTTAGGTGACTTACGGTCCCTTGAAAATCTCCAAGAGGCTATCGTCAAGATGTCCATGATTAGCTCTAAGGTTATCGGCTTAGTGAATCCTGCTGGTATCACCCAGCCACGCCGACTGACCAAAGCTCAGACTGGTGACTTCGTTACTGGTCGTCCAGAAGACATCTCGTTCCTCCAACTGGAGAAGCAAGTAGACTTTACTGTAGCTAACGc                                                                                                                                                                                                                                                                                                         <  1:762031/291‑1 (MQ=255)`  
    `tATCCTAAAGAGGCTTGCCCATACATCCCGATTCGGATGGTCAGACTAGATGGTGAATCCTACGGTCGTTCGTACATTGAGGAATACTTAGGTGACTTACGGTCCCTTGAAAATCTCCAAGAGGCTATCGTCAAGATGTCCATGATTAGCTCTAAGGTTATCGGCTTAGTGAATCCTGCTGGTATCACCCAGCCACGCCGACTGACCAAAGCTCAGACTGGTGACTTCGTTACTGGTCGTCTAGAAGACATCTCGTTCCTCCAACTGGAGAAGCAAGCAGACTTTACTGTAGCTAACGcct                                                                                                                                                                                                                                                                                                       >  1:100058/1‑300 (MQ=255)`  
     `aTCCTAAAGAGGCTTGCCCATACATCCCGATTCGGATGGTCAGACTAGATGGTGAATCCTACGGTCGTTCGTACATTGAGGAATACTTAGGTGACTTACGGTCCCTTGAAAATCTCCAAGAGGCTATCGTCAAGATGTCCATGATTAGCTCTAAGGTTATCGGCTTAGTGAATCCTGCTGGTATCACCCAGCCACGCCGACTGACCAAAGCTCAGACTGGTGACTTCGTTACTGGTCGTCCAGAAGACATCTCGTTCCTCCAACTGGAGAAGCAAGCAGACTTTACTGTAGCTAACGCCGt                                                                                                                                                                                                                                                                                                      >  1:384779/1‑301 (MQ=255)`  
          `gtgggctcggagatgtgtataagagacaggATGGTCAGACTAGATGGTGAATCCTACGGTCGTTCGTACATTGAGGAATACTTAGGTGACTTACGGTCCCTTGAAAATCTCCAAGAAGCTATCGTCAAGATGTCCATGATTAGCTCTAAGGTTATCGGCTTAGTGAATCCTGCTGGTATCACCCAGCCACGCCGACTGACCAAAGCTCAGACTGGTGACTTCGTTACTGGTCGTCCAGAAGACATCTCGTTCCTCCAACTGGAGAAGCAAGCAGACTTTACTGTAGCTAACGCCGTAAGTg                                                                                                                                                                                                                                                                                                 <  1:674006/273‑1 (MQ=255)`  
             `gagGCTTGCCCATACATCCCGATTCGGATGGTCAGACTAGATGGTGAATCCTATGGTCGTTCGTACATTGAGGAATACGTAGGTGACTTACGGTCCCTTGAAAATCTCCAAGAGGCTATCGTCAAGATGTCCATGATCAGCTCTAAGGTTATCGGCTTAGGGAAGCCTGCTGGTATCACCCAGCCACGCCGACTGACCAAAGCTCAGACTGGTGACTTCGGTACTGGTCGTCCAGAAGACATCTCGTTCCTCCAACTGGAGAAGCAAGCAGACTTTACTGTAGCTAACGCCGTAAGTGACg                                                                                                                                                                                                                                                                                              <  1:810277/301‑1 (MQ=255)`  
               `ggCTTGCCCATACATCCCGATTCGGATGGTCAGACTAGATGGTGAATCCTACGGTCGTTCGTACATTGAGGAATACTTAGGTGACTTACGGTCCCTTGAAAATCTACAAGAGGCTATCGTCAAGATGTCCATGATTAGCTCTAAGGTTATCGGCTTAGTGAATCCTGCTGGTATCACCCAGCCACGCCGACTGACCAAAGCTCAGACTGGTGACTTCGTTACTGGTCGTCCAGAAGACATCTCGTTCCTCCAACTGGAGAAGCAAGCAGACTTTACTGTCGCTAACGCCTTAAGTGACCCt                                                                                                                                                                                                                                                                                            >  1:488941/1‑301 (MQ=255)`  
                 `cTTGCCCTTACATCCCGATTCGGATGGTCAGACTAGATGGTGAATCCTACGGTCGTTCGTACATTGAGGACTACTTAGGTGACTTACGGTCCCTTGAAAATCTCCAAGAGGCTATCGTCAAGATGTCCATGATTAGCTCTAAGGTTATCGGCTTAGTGAATCCTGCTGGTATATCCCAGCCACGCCGACTGACCAAAGCTCAGACTGGTGACTTCGTTACTGGTCGTCCAGAAGACATCTCGTTCCTCCAACTGGAGAAGCAAGCAGACTTTACTGTAGCTAACGCCGTAAGTGACGCTAt                                                                                                                                                                                                                                                                                          <  1:86104/301‑1 (MQ=255)`  
                   `tGCCCATACATCCCGATTCGGATGGTCAGACTAGATGGTGAATCCTACGGTCGTTCGTACATTGAGGAATACTTAGGTGACTTACGGTCCCTTGAAAATCTCCAAGAGGCTATCGTCAAGATGTCCATGATTAGCTCTAAGGTTATCGGCTTAATGAATCCTTCTGGTATCACACAGCCACGCCGACTGACCAAAGCTCAGACTGGTTACTTCGTTACTGGTCGTCCAGCAGACATCTCGTTCCTCCAACTGGTGAAGCAAGCAGACTGTACTGTAGCTAACACCGTAAGTGCCGCTATCg                                                                                                                                                                                                                                                                                        >  1:717974/1‑301 (MQ=255)`  
                                 `gATTCGGATGGTCAGACTAGATGGTGAATCCTACGGTCGTTCGTACATTGAGGAATACTTAGGTGACTTACGGTCCCTTGAAAATCTCCAAGAGGCTATCGTCAAGATGTCCATGATTAGCTCTAAGGTTATCGGCTTAGTGAATCCTGCTGGTATCACCCAGCCACGCCGACTGACCAAAGCTCAGACTGGTGACTTCGTTACTGGTCGTCCAGAAGACATCTCGTTCCTCCAACTGGAGAAGCAAGCAGACTTTACTGTAGCTAACGCCGTAAGTGACGCTATTGCGGCTCGCCTTTCg                                                                                                                                                                                                                                                                          >  1:331721/1‑301 (MQ=255)`  
                                     `cGGATGGTCAGACTAGATGGTGAATCCTACGGTCGTTCGTACATTGAGGAATACTTAGGTGACTTACGGTCCCTTGAAAATCTCCAAGAGGCTATCGTCAAGATGTCCATGATTAGCTCTAAGGTTATCGGCTTAGTGAATCCTGCTGGTATCACCCAGCCACGCCGACTGACCAAAGCTCAGACTGGTGACTTCGTTACTGGTCGTCCAGAAGACATCTCGTTCCTCCAACTGGAGAAGCAAGCAGACTTTACTGTAGCTAACGCCGTAAGTgctgtctcttatacacatctccgagccc                                                                                                                                                                                                                                                                      >  1:578267/1‑274 (MQ=255)`  
                                         `tGGTCAGACTAGATGGTGAATCCTACGGTCGTTCGTACATTGAGGAATACTTAGGTGACTTACGGTCCCTTGAAAATCTCCAAGAGGCTATCGTCAAGATGTCCATGATTAGCTCTAAGGTTATCGGCTTAGTGAATCCTGCTGGTATCACCCAGCCACGCCGACTGACCAAAGCTCAGACTGGTGACTTCGTTACTGGTCGTCCAGAAGACATCTCGTTCCTCCAACTGGAGAAGCAAGCAGACTTTACTGTAGCTAACGCCGTAAGTGCCGCTATCGAGGCTCGCCTTTCGTTTGCCtt                                                                                                                                                                                                                                                                  >  1:286937/1‑301 (MQ=255)`  
                                          `ggTCAGACTAGATGGTGAATCCTACGGTCGTTCGTACATTGAGGAATACTTAGGTGACTTACGGTCCCTTGAAAATCTCCAAGAGGCTATCGTCAAGATGTCCATGATTAGCTCTAAGGTTATCGGCTTAGTGAATCCTGCTGGTATCACCCAGCCACGCCGACTGACCAAAGCTCAGACTGGTGACTTCGTTACTGGTCGTCCAGAAGACATCTCGTTCCTCCAACTGGAGAAGCAAGCAGACTTTACTGTAGCTAACGCCGTAAGTTACGCTATCGCGGCTCGCCTTTCGTTTGCCtgt                                                                                                                                                                                                                                                                 >  1:630945/1‑299 (MQ=255)`  
                                              `tgtataagagacagaTCCTACGGTCGTTCGTACATTGAGGAATACTTAGGTGACTTACGGTCCCTTGAAAATCTCCAAGAGGCTATCGTCAAGATGTCCATGATTAGCTCTAAGGTTATCGGCTTAGTGAATCCTGCTGGTATCACCCAGCCACGCCGACTGACCAAAGCTCAGACTGGTGACTTCGTTACTGGTCGTCCAGAAGACATCTAGTTCTTCCAACTGGAGAAGCAAGCAGACTTTACTGTAGCTAACGCCGTAAGTGACGCTATCGCGGCTCGCCTTTCGTTTGCCTTTATGt                                                                                                                                                                                                                                                             <  1:293791/287‑1 (MQ=255)`  
                                              `aGACTAGATGGTGAATCCTACGGTCGTTCGTACATTGAGGAATACTTAGGTGACTTACGGTCCCTTGAAAATCTCCAAGAGGCTATCGTCAAGATGTCCATGATTAGCTCTAAGGTTATCGGCTTAGTGAATCCTGCTGGTATCACCCAGCCACGCCGACTGATCAAAGCTCAGACTGGTGACTTCGTTACTGGTCGTCCAGAAGACATCTCGTTCCTCCAACTGGAGAAGCAAGCAGACTTTACTGTAGCTAACGCCGTAAGTGACGCTATCGCGGCTCGCCTTTCGTTTGCCTTTATGt                                                                                                                                                                                                                                                             <  1:339409/301‑1 (MQ=255)`  
                                              `aGACTAGATGGTGAATCCTACGGTCGTTCGTACATTGAGGAATACTTAGGTGACTTACGGTCCCTTGAAAATCTCCAAGAGGCTATCGTCAAGATGTCCATGATTAGCTCTAAGGTTATCGGCTTAGTGAATCCTGCTGGTATCACCCAGCCACGCCGACTGACCAAAGCTCAGACTGGTGACTTCGTTACTGGTCGTCCAGAAGACATCTCGTTCCTCCAACTGGAGAAGCAAGCAGACTTTACTGTAGCTAACGCCGTAAGTGACGCTATCGCGGCTCGCCTTTCGTTTGCCTTTATGt                                                                                                                                                                                                                                                             <  1:452319/301‑1 (MQ=255)`  
                                               `gACTTGATGGTGAATCCTACGGTCGTTCGTACATTGAGGAATACTTAGGTGACTTACGGTCCCTTGAAAATATCCAAGAGGCTATCGTCAAGATGTCCATGTTTAGCTCTAAGGTTATCGGCTTAGTGAATCCTGCTGGTATCACCCAGCCACGCCGACTGACCAAAGCTCAGACTGGTGACTTCGTTACTGGTCGTCCAGAAGACATCTCGTTCCTCCAACTGGTGAAGCAAGCAGACTTTACTGTTGCTAACGCCGTAAGTGACGCTATCGCGGCTCGCCTTTCGTTTGCCTTTATGtt                                                                                                                                                                                                                                                            >  1:207771/1‑301 (MQ=255)`  
                                                   `ggctcggagatgtgtataagagacagaCATTGAGGAATACGTAGGTGACTTACGGTCCCTTGAAAATCTCCAAGAGGCTATCGTCAAGATGTCCATGATTAGCTCTAAGGTTATCGGCTTAGTGAATCCTGCTGGTATCACCCAGCCACGCCGACTGACCAAAGCTCAGACTGGTGACTTCGTTACTGGTCGTCCAGAAGACATCTCGTTCCTCCAACTGGAGAAGCAAGCAGACTTTACTGTAGCTAACGCCGTAAGTGACGCTATCGCGGCTCGCCTTTCGTTTGCCTTTATGTTGAAc                                                                                                                                                                                                                                                        <  1:192883/275‑1 (MQ=255)`  
                                                        `gTGAATCCTACGGTCGTTCGTACATTGAGGAATACTTAGGTGACTTACGGTCCCTTGAAAATCTCCAAGAGGCTATCGTCAAGATGTCCATGATTAGCTCTAAGGTTATCGGCGTAGTGAATCCTGCTGGTATCACCCAGCCACGCCGACTGACCAAAGCTCAGACTGGTGACTTCGTTACTGGTCGTCCAGAAGACATCTCGTTCCTCCAACTGGAGAAGCAAGCAGACTTTACTGTAGCTAACGCCGTAAGTGCCGCTATCGAGGCTCGCCTTTCGTTTGCCTTTATGTTGAACTCTGc                                                                                                                                                                                                                                                   <  1:134253/301‑1 (MQ=255)`  
                                                            `aTCCTACGGTCGTTCGTACATTGAGGAATACTTAGGTGACTTACGGTCCCTTGAAAATCTCCAAGAGGCTATCGTCAAGATGTCCATGATTAGCTCTAAGGTTATCGGCTTAGTGAATCCTGCTGGTATCACCCAGCCACGCCGACTGACCAAAGCTCAGACTGGTGACTTCGTTACTGGTCGTCCAGAAGACATCTCGTTCCTCCAACTGGAGAAGCAAGCAGACTTTACTGTAGCTAACGCCGTAAGTGACGCTATCGCGGCTCGCCTTTCGTTTGCATTTATGTTGAActcctgtctc                                                                                                                                                                                                                                               >  1:355335/1‑294 (MQ=255)`  
                                                             `tCCTACGGTCGTTCGTACATTGAGGAATACTTAGGTGACTTACGGTCCCTTGAAAATCTCCAAGAGGCTATCGTCAAGATGTTCATGATTAGCTCTAAGGTTATCGGCTTAGTGAATCTTGCTGGTATCACCCAGCCACGCCGACTGACCAAAGCTCAGACTGGTGACTTCGCTACTTGTCGTCCGGAAGACATCTCGTTACTCTAACTGGAGTAGTAGGCAGACTTTACTGTAGCTAATGCTCTAACTGAAGCTATCGCCACTCGCTGTTTGTTTGCGTTTACTTTGAACCCTGCGGttt                                                                                                                                                                                                                                              >  1:169902/1‑300 (MQ=255)`  
                                                                    `agcgttcgtACATTGAGGAATACTTAGGTGACTTACGGTCCCTTGAAAATCTCCAAGAGGCTATCGTCAAGATGTCCATGATTAGCTCTAAGGTTATCGGCTTAGTGAATCCTGCTGGTATCACCCAGCCACGCCGACTGACCAAAGCTCAGACTGGTGACTTCGTTACTGGTCGTCCAGAAGACATCTCGTTCCTCCAACTGGAGAAGCAAGCAGACTTTACTGTAGCTAACGCCGTAAGTGACGCTATCGCGGCTCGCCTTTCGTTTGCCTTTATGTTGAACTCTGCGGTTCAGCGTAc                                                                                                                                                                                                                                       <  1:249086/299‑1 (MQ=255)`  
                                                                         `tagtAAATTGAGGAATACTTAGGTGACTTACGGTACCTTGAAAATCTGCAAGAGGCTATCGTCAAGATGTCCATGATTAGCTCTAAGGTTATCGGCTTAGTGAATCCTGCTGGTATCACCCAGCCACGCCGACTGACCAAAGCTCAGACTGGTGACTTCGTTACTGGTCGTCCAGAAGACATCTCGTTCCTCCAACTGGAGAAGCAAGCAGACTTTACTGTAGCTAACGCCGTAAGTGACGCTATCGCGGCTCGCCTTTCGTTTGCCTTTATGTTGAACTCTGCGGTTCAGCGTACAGGTg                                                                                                                                                                                                                                  <  1:504677/299‑1 (MQ=255)`  
                                                                              `cATTGAGTAATACTTAGGTGACTTACGGTCCCTTGAAAATCTCCAAGAGGCTATCGTCAAGATGTCCATGATTAGCTCTAAGGTTATCGGCTTAGTGAATCCTGCTGGTATCACCCAGCCACGCCGACTGACCAAAGCTCAGACTGGTGACTTCGTTACTGGTCGTCCAGAAGACATCTCGTTCCTCCAACTGGAGAAGCAAGCAGACTTTACTGTAGCCAAAGCCGTAAGTGACGCTATCGCGGCTCGCCTTTCGTTTGCATTTATGTTGAAATCTGCGGTTAAGCGTACAggctgtctt                                                                                                                                                                                                                             >  1:108117/1‑294 (MQ=255)`  
                                                                              `cATTGAGGAATACTTAGGTGACTTACGGTCCCTTGAAAATCTCCAAGAGGCTATCGTCAAGATGTCCATGATTAGCTCTAAGGTTATCGGCTTAGTGAATCCTGCTGGTATCACCCAGCCACGCCGACTGACCAAAGCTCAGACTGGTGACTTCGTTACTGGTCGTCCAGAAGACATCTCGTTCCTCCAACTGGAGAAGCAAGCAGACTTTACTGTAGCTAACGCCGTAAGTGACGCTATCGCGGCTCGCCTTTCGTTTGCCTTTATGTTGAACTCTGCGGTTCAGCGTACAGGTGAACgt                                                                                                                                                                                                                             <  1:308710/301‑1 (MQ=255)`  
                                                                              `agttGAAGAATAATTAGGTGACTTACGGTCCCTTGAAAATCTCCAAGAGGATAACGTCAAGATGTCCATGATTAGCTCTAAGGTTATCGGCTTAGTGAATCATGCTGGTATCACCCAGCCACGCCGACTGACCAAAGCTCAGACTGGTGACTTCGTTACTGGTCGTCCAGAAGACATCTCGTTCCTCCAACTGGAGAAGCAAGCAGACTTTACTGTAGCTAACGCCGTAAGTGACGCTATCGCGGCTCGCCTTTCGTTTGCCTTTATGTTGAACTCTGCGGTTCAGCGTACAGGTGAACgt                                                                                                                                                                                                                             <  1:148348/299‑1 (MQ=255)`  
                                                                                 `tGAGGAATACTTAGGTGACTTACGGTCCCTTGAAAATCTCCAAGAGGCTATCGTCAAGATGTCCATGATTAGCTCTAAGGTTATCGGCTTAGTGAATCCTGCTGGTATCACCCAGCCACGCCGACTGACCAAAGCTCAGACTGGTGACTTCGTTACTGGTCGTCCAGAAGACATCTCGTTCCTCCAACTGGAGAAGCAAGCAGACTTTACTGTAGCTAACGCCGTAAGTGACGCTATCGCGGCTCGCCTTTCGTTTGCCTTTATGTTGAACTCTGCGGTTCAGCGTACAGGTGAACGtgtg                                                                                                                                                                                                                          <  1:423736/301‑1 (MQ=255)`  
                                                                                   `gggAATACTTAGGTAACTTACGGTCCCTTGAAAATCTCCAAGAGGCTATCGTCAAGATGTCCATGATTAGCTCTAAGGTTATCGGCTTAGTGAATCCTGCTGGTATCACCCAGCCACGCCGACTGACCAAAGCTCAGACTGGTGACTTCGTTACTGGTCGTCCAGAAGACATCTCGTTCCTCCAACTGGAGAAGCAAGCAGACTTTACTGTAGCTAACGCCGTAAGTGACGCTATCGCGGCTCGCCTTTCGTTTTTTTTTATGTTGCCCTCTGCGGTTCAGCGTACAGGTGAACGTGTGAc                                                                                                                                                                                                                        <  1:392472/300‑1 (MQ=255)`  
                                                                                   `atgtgtataagagacagttACGGTCCCTTGAAAATCTCCAAGAGGCTATCGTCAAGATGTCCATGATTAGCTCTAAGGTTATCGGCTTAGTGAATCCTGCTGGTATCACCCAGCCACGCCGACTGACCAAAGCTCAGACTGGTGACTTCGTTACTGGTCGTCCAGAAGACATCTCGTTCCTCCAACTGGAGAAGCAAGCAGACTTTACTGTAGCTAACGCCGTAAGTGACGCTATCGCGGCTCGCCTTTCGTTTGCCTTTATGTTGAACTCTGCGGTTCAGCGTACAGGTGAACGTGTGAc                                                                                                                                                                                                                        <  1:11609/284‑1 (MQ=255)`  
                                                                                    `cagaTACTTAGGTGACTTACGGTCCCTTGAAAATCTCCAAGAGGCTATCGTCAAGATGTCCATGATTAGCTCTAAGGTTATCGGCTTAGTGAATCCTGCTGGTATCACCCAGCCACGCCGACTGACCAAAGCTCAGACTGGTGACTTCGTTACTGGTCGTCCAGAAGACATCTCGTTCCTCCAACTGGAGAAGCAAGCAGACTTTACTGTAGCTAACGCCGTAAGTGACGCTATCGCGGCTCGCCTTTCGTTTGCCTTTATGTTGAACTCTGCGGTTCAGCGTACAGGTGAACGTGTGAcc                                                                                                                                                                                                                       <  1:232369/298‑1 (MQ=255)`  
                                                                                       `aTACTTAGGTGACTTACGGTCCCTTGAAAATCTCCAAGAGGCTATCGTCAAGATGTCCATGACTAGTTCTAAGGTTATGGGCTTAGTGAATCCTGCTGGTATTACCCAGCCACGCCGACTGACTAAAGCTCAGACTGGTGACTTCGTTAATGGTCGTCCTGAAGACAGCCCGTTCCGCCGACCGCAGAAGCAGGCAGACTTCACTGTAGATAACGCTGCAGGTGACGTTACCGCGGCTCGTCTTTCGTTTGCCTTTATGTTGAACTCTGCGGTTCTCCGTatgggttaatgcgttgcagcc                                                                                                                                                                                                                    >  1:802499/1‑281 (MQ=255)`  
                                                                                         `aCTTAGGTGACTTACGGTCCCTTGAAAATCTCCAAGAGGCTATCGTCAAGATGTCCATGATTAGCTCTAAGGTTATCGGCTTAGTGAATCCTGCTGGTATCACCCAGCCACGCCGACTGACCAAAGCTCAGACTGGTGACTTCGTTACTGGTCGTCCAGAAGACATCTCGTTCCTCCAACTGGAGAAGCAAGCAGACTTTACTGTAGCTAACGCCGTAAGTGACGCTATCGCGGCTCGCCTTTCGTTTGCCTTTATGTTGAACTCTGCGGTTCAGCGTACAGGTGAACGTGTGACCGCCGa                                                                                                                                                                                                                  >  1:140206/1‑301 (MQ=255)`  
                                                                                         `aCTTAGGTGACTTACGGTCCCTTGAAAATCTCCAAGAGGCTATCGTCAAGATGTCCATGATTAGCTCTAAGGTTATCGGCTTAGTGAATCCTGCTGGTATCACCCAGCCACGCCGACTGACCAAAGCTCAGACTGGTGACTTCGTTACTGGTCGTCCAGAAGACATCTCGTTCCTCCAACTGGAGAAGCAAGCAGACTTTACTGTAGCTAACGCCGTAAGTGACGCTATCGAGGCTCGCCTTTCGTTTGCCTTTATGTTGAACTCTGCGGTTCAGCGTCCAGGTGAACGAGTGACCGCCGa                                                                                                                                                                                                                  >  1:467625/1‑301 (MQ=255)`  
                                                                                          `cTTAGGTGACTTACGGTCCCTTGAAAATCTCCAAGAGGCTATCGTCAAGATGTCCATGATTAGCTCTAAGGTTATCGGCTTAGTGAATCCTGCTGGTATCACCCAGCCACGCCGACTGACCAAAGCTCAGACTGGTGACTTCGTTACTGGTCGTCCAGAAGACATCTCGTTCCTCCAACTGGAGAAGCAAGCAGACTTTACTGTAGCTAACGCCGTAAGTGACGCTATCGCGGCTCGCCTTTCGTTTGCCTTTATGTTGAACTCTGCGGTTCAGCGTACAGGTGAACGTGTGACCGCCGaa                                                                                                                                                                                                                 >  1:601861/1‑301 (MQ=255)`  
                                                                                                 `gACTTACGGTCCCTTGAAAATCTCCAAGAGGCTATCGTCAAGATGTCCATGATTAGCTCTAAGGTTATCGGCTTAGTGAATCCTGCTGGTATCACCCAGCCACGCCGACTGACCAAAGCTCAGACTGGTGACTTCGTTACTGATCGTCCAGAAGACATCTCGTTCCTCCAACTGGAGAAGCAAGCAGACTTTACTGTAGCTAACGCCGTAAGTGACGCTATCGCGGCTCGCCTTTCGTTTGCCTTTATGTTGAACTCTGCGGTTCAGCGTACAGGTGAACGTGTGCCCGCCGAAGAGATTc                                                                                                                                                                                                          >  1:585844/1‑301 (MQ=255)`  
                                                                                                   `cTTATGGTCCCTTGAAAATCTCCAAGAGGCTATCGTCAAGATGTCCATGATTAGCTCTAAGGTTATCGGCTTAGTGAATCCTGCTGGTATCACCCAGCCACGCCGACTGACCAAAGCTCAGACTGGTGACTTCGTTACTGGTCGTCCAGAAGACCTCTCGTTCCTCCAACTGGAGAAGCAAGCAGACTTTACTGTAGCTAACGCCGTAAGTGACGCTATCGCGGCTCGCCTTTCGTTTGCCTTTATGTTGAACTCTGCGGTTCAGCGTACAGGTGAACGTGTGACCGCCGAAGAGATTCgg                                                                                                                                                                                                        >  1:154973/1‑301 (MQ=255)`  
                                                                                                   `cTTACGGTCCCTTGAAAATCTCCAAGATGCTATCGTCAAGATGTCCATGATTAGCTCTAAGGTTATCGGCTTAGTGAATCCTGCTGGTATCACCCAGCCACGCCGACTGACCAAAGCTCAGACTGGTCACTTCGTTACTGGTCGTCCAGAAGACATCTCGTTCCTCCAACTGGAGCAGCAAGCTGACTTTACCGTAGCTAACGCCGTAAGTGACACTATCGCGGCCTGATATTCGTTTGCCTTTATGTTTAACTCGGCGTTTCACCTTTCAGGTGTACGTCTAACCGCCGCAGTGATTCgg                                                                                                                                                                                                        >  1:795088/1‑301 (MQ=255)`  
                                                                                                           `cccTTGAAAATCTCCAAGAGGCTATCGTCAAGATGTCCATGATTAGCTCTAAGGTTATCGGCTTAGTGAATCCTGCTGGTATCACCCAGCCACGCCGACTGACCAAAGCTCAGACTGGTGACTTCGTTACTGGTCGTCCAGAAGACATCTCGTTCCTCCAACTGGAGAAGCAAGCAGACTTTACTGTAGCTAACGCCGTAAGTGCCGCTATCGAGGCTCGCCTTTCGTTTGCCTTTATGTTGAACTCTGCGGTTCAGCGTACAGGTGAACGTGTGACCGCCGCAGAGATTCGGTATGTAGc                                                                                                                                                                                                >  1:179653/1‑301 (MQ=255)`  
                                                                                                           `cccTTGAAAATCTCCAAGAGGCTATCGTCAAGATGTCCATGATTAGCTCTAAGGTTATCGGCTTAGTGAATCCTGCTGGTATCACCCAGCCACGCCGACTGACCAAAGCTCAGACTGGTGACTTCGTTACTGGTCGTCCAGAAGACATCTCGTTCCTCCAACTGGAGAAGCAAGCAGACTTTACTGTAGCTAACGCCGTAAGTGACGCTATCGCGGCTCGCCTTTCGTTTGCCTTTATGTTGAACTCTGCGGTTCAGCGTACAGGTGAACGTGTGACCGCCGAAGAGATTCGGTATGTAGc                                                                                                                                                                                                <  1:824345/301‑1 (MQ=255)`  
                                                                                                             `cTTGAAAATCTCCAAGAGGCTATCGTCAAGATGTCCATGATTAGCTCTAAGGTTATCGGCTTAGTGAATCCTGCTGGTATCACCCAGCCACGCCGACTGACCAAAGCTCAGACTGGTGACTTCGTTACTGGTCGTCCAGAAGACATCTCGTTCCTCCAACTGGAGAAGCAAGCAGACTTTACTGTAGCTAACGCCGTAAGTGACGCTATCGCGGCTCGCCTTTCGTTTGCCTTTATGTTGAACTCTGCGGTTCAGCGTACAGGTGAACGTGTGACCGCCGAAgagactgtctcttatacac                                                                                                                                                                                              >  1:369466/1‑286 (MQ=255)`  
                                                                                                                  `aaaTCTCAAAGAGGCTATCGTCAAGATGTCCATGATTAGCTCTAAGGTTATCGGCTTAGTGAATCCTGCTGGTATCACCCAGCCACGCCGACTGACCAAAGCTCAGACTGGTGACTTCGTTACTGGTCGTCCAGAAGACATCTCGTTCCTCCAACTGGAGAAGCAAGCAGACTTTACTGTAGCTAACGCCGTAAGTGACGCTATCGCGGCTCGCCTTTCGTTTGCCTTTATGTTGAACTCTGCGGTTCAGCGTACAGGTGAACGTGTGACCGCCGAAGAGATTCGGTATGTAGCTTCTGaa                                                                                                                                                                                         <  1:460593/301‑1 (MQ=255)`  
                                                                                                                    `aTCTCCAAGAGGCTATCGTCAAGATGTCCATGATTAGCTCTAAGGTTATCGGCTTAGTGAATCCTGCTGGTATCACCCAGCCACGCCGACTGACCAAAGCTCAGACTGGTGACTTCGTTACTGGTCGTCCAGAAGACATCTCGTTCCTCCAACTGGAGAAGCAAGCAGACTTTACTGTAGCTAACGCCGTAAGTGACGCTATCGCGGCTCGCCTTTCGTTTGCCTTTATGTTGAACTCTGCGGTTCAGCGTACAGGTGAACGTGTGACCGCCGAAGAGATTCGGTATGTAGCTTCTTAACt                                                                                                                                                                                       >  1:265158/1‑301 (MQ=255)`  
                                                                                                                                   `tCGTCAAGATGTCCATGATTAGCTCTAAGGTTATCGGCTTAGTGAATCCTGCTGGTATCACCCAGCCACGCCGACTGACCAAAGCTCAGACTGGTGACTTCGTTACTGGTCGTCCAGAAGACATCTCGTTCCTCCAACTGGAGAAGCAAGCAGACTTTACTGTAGCTAACGCCGTAAGTGCCGCTATCGAGGCTCGACTTTCGTTTGCCTTTATGTTGAACTCTGCGGTTCAGCGTACAGGTGAACGTGTGACCGCCGAAGAGATTCGGTATGTAGCTTCTGAACTTGAAGATACTTTAgg                                                                                                                                                                        <  1:257892/301‑1 (MQ=255)`  
                                                                                                                                   `tCGTCAAGATGTCCATGATTAGCTCTAAGGTTATCGGCTTAGTGAATCCTGCTGGTATCACCCAGCCACGCCGACTGACCAAAGCTCAGACTGGTGACTTCGTTACTGGTCGTCCAGAAGACATCTCGTTCCTCCAACTGGAGAAGCAAGCAGACTTTACTGTAGCTAACGCCGTAAGTGACGCTATCGCGGCTCGCCTTTCGTTTGCC‑TTATGTTGAACTCTGCGGTTCAGCGTACAGGTGAACGTGTGACCGCCGAAGAGATTCGGTATGTAGCTTCTGAACTTGAAGATACTTTAggt                                                                                                                                                                       <  1:624205/301‑1 (MQ=255)`  
                                                                                                                                    `cGTCAAGATGTCCATGATTAGGTCTAAGGTTATCGGCTTAGTGAATCCTGCTGGTATGCCCCAGCCACGCCGACTGACAATAGCACAGACTGGTGACTTCGTTACAGTACGTCCAGAAGACATCTCGTTCCTCCAACTGGAGAAGCAAGCAGACTTTACTTTAGCTAACGCCGTAAGTGCCGCTATCGAGGCTCGCCTTTCGTTTGCCTTTATGTTGAACTCTGCGGTTCAGCGTACAGGTGAACGTGTGACCGCCGAAGAGATTCGGTATGTAGCTTCTGAACTTGAAGATACTTTAggt                                                                                                                                                                       <  1:163600/301‑1 (MQ=255)`  
                                                                                                                                      `tCAAGATGTCCATGATTAGCTCTAAGGTTATCGGCTTAGTGAATCCTGCTGGTATCACCCAGCCACGCCGACTGATCAAAGCTCAGACTGGTGACTTCGTTACTGGTCGTCCAGAAGACATCTCGTTCCTCCAACTGGAGAAGCAAGCAGACTTTACTGTAGCTAACGCCGTAAGTGACGCTATCGCGGCTCGCCTTTCGTTTGCCTTTATGTTGAACTCTGCGGTTCAGCGTACAGGTGAACGTGTGACCGCCGAAGAGATTCGGTATGTAGCTTCTGAACTTGAAGATACTTTAggtgg                                                                                                                                                                     <  1:555674/301‑1 (MQ=255)`  
                                                                                                                                      `tCAAGATGTCCATGATTAGCTCTAAGGTTATCGGCTTAGTGAATCCTGCTGGTATCACCCAGCCACGCCGACTGACCAAAGCTCAGACTGGTGACTTCGTTACTGGTCGTCCAGAAGACATCTCGTTCCTCCAACTGGAGAAGCAAGCAGACTTTACTGTAGCTAACGCCGTAAGTGACGCAATCGCGGCTCGCCTTTCGTTTGCCTTTATGTTGAACTCTGCGGTTCAGCGTACAGGTGAACGTGTGACCGCCGAAGAGATTCGGTATGTAGCTTCTGAACTTGAAGATACTTTAggtgg                                                                                                                                                                     <  1:594058/301‑1 (MQ=255)`  
                                                                                                                                       `cAAGATGTCCATGATTAGCTCTAAGGTTATCGGCTTAGTGAATCCTGCTGGTATCACCCAGCCACGCCGACTGACCAAAGCTCAGACTGGTGACTTCGTTACTGGTCGTCCAGAAGACATCTCGTTCCTCCAACTGGAGAAGCAAGCAGACTTTACTGTAGCTAACGCCGTAAGTGACGCTATCGCGGCTCGCCTTTCGTTTGCCTTTATGTTGAACTCTGCGGTTCAGCGTACAGGTGAACGTGTGACCGCCGAAGAGATTCGGTATGTAGCTTCTGAACTTGAAGATACTTTAggtggt                                                                                                                                                                    <  1:649282/301‑1 (MQ=255)`  
                                                                                                                                              `tCCATGATTAGCTCTAAGGTTATCGGCTTAGTGAATCCTGCTGGTATCACCCAGCCACGCCGACTGACCAAAGCTCAGACTGGTGACTTCGTTACTGGTCGTCCAGAAGACATCTCGTTCCTCCAACTGGAGAAGCAAGCAGACTTTACTGTAGCTAACGCCGTAAGTGACGCTATCGCGGCTCGCCTTTCGTTTGCCTTTATGTTGAACTCTGCGGTTCAGCGTACAGGTGAACGTGTGACCGCCGAAGAGATTCGGTATGTCGCTTCTGAACTTGAAGATACTTTAGGTGGTGTCTAct                                                                                                                                                             >  1:325282/1‑301 (MQ=255)`  
                                                                                                                                               `ccATGATTAGCTCTAAGGTTATCGGCTTAGTGAATCCTGCTGGTATCACCCAGCCACGCCGACTGACCAAAGCTCAGACTGGTGACTTCGTTACTGGTCGTCCAGAAGACATCTCGTTCCTCCAACTGGAGAAGCAAGCAGACTTTACTGTAGCTAACGCCGTAAGTGACGCTATCGCGGCTCGCCTTTCGTTTGCCTTTATGTTGAACTCTGCGGTTCAGCGTACAGGTGAACGTGTGACCGCCGAAGAGATTCGGTATGTAGCTTCTGAACTTGAAGATACTTTAGGTGGTGTCTActc                                                                                                                                                            <  1:789751/301‑1 (MQ=255)`  
                                                                                                                                               `ccATGATTAGCTCTAAGGTTATCGGCTTAGTGAATCCTGCTGGTATCACCCAGCCACGCCGACTGACCAAAGCTCAGACTGGTGACTTCGTTACTGGTCGTCCAGAAGACATCTCGTTCCTCCAACTGGAGAAGCAAGCAGACTTTACTGTAGCTAACGCCGTAAGTGACGCTATCGCGGCTCGCCTTTCGTTTGCCTTTATGTTGAACTCTGCGGTTCAGCGTACAGGTGAACGTGTGACCGCCGAAGAGATTCGGTATGTAGCTTCTGAACTTGAAGATACTTTAGGTGGTGTCTActc                                                                                                                                                            <  1:540831/301‑1 (MQ=255)`  
                                                                                                                                               `ccATGATTAGCTCTAAGGTTATCGGCTTAGTGAATCCTGCTGGTATCACCCAGCCACGCCGACTGACCAAAGCTCAGACTGGTGACTTCGTTACTGGTCGTCCAGAAGACATCCCGTTCCTCCAACTGGAGAAGCAAGCAGACTTTACTGTAGCTAACGCCGTAAGTGACGCTATCGCGGCTCGCCTTTCGTTTGCCTTTATGTTGAACTCTGCGGTTCAGCGTACAGGTGAACGTGTGACCGCCGAAGAGATTCGGTATGTAGCTTCTGAACTTGAAGATACTTTAGGTGGTGTCTActc                                                                                                                                                            <  1:346774/301‑1 (MQ=255)`  
                                                                                                                                                    `tgtataagagacagtATCGGCTTAGTGAATCCTGCTGGTATCACCCAGCCACGCCGACTGACCAAAGCTCAGACTGGTGACTTCGTTACTGGTCGTCCAGAAGACATCTCGTTCCTCCAACTGGAGAAGCAAGCAGACTTTACTGTAGCTAACGCCATAAGTGACGCTATCGCGGCTCGCCTTTCGTTTGCCTTTATGTTGAACTCTGCGGTTCAGCGTACAGGTGAACGTGTGACCGCCGAAGAGATTCGGTATGTAGCTTCTGAACTTGAAGATACTTTAGGTGGTGTCTACTCTATcc                                                                                                                                                       <  1:121839/287‑1 (MQ=255)`  
                                                                                                                                                    `ggggctcggagatgtgtataagagacagaTCCTGCTGGTATCACCCAGCCACGCCGACTGACCAAAGCTCAGACTGGTGACTTCGTTACTGGTCGTCCAGAAGACATCTCGTTCCTCCAACTGGAGAAGCAAGCAGACTTTACTGTAGCTAACGCCGTAAGTGACGCTATCGCGGCTCGCCTTTCGTTTGCCTTTATGTTGAACTCTGCGGTTCAGCGTACAGGTGAACGTGTGACCGCCGAAGAGATTCGGTATGTAGCTTCTGAACTTGAAGATACTTTAGGTGGTGTCTACTCTATcc                                                                                                                                                       <  1:704517/273‑1 (MQ=255)`  
                                                                                                                                                       `aGCTCTAAGGTTATCGGCTTAGTGAATCCTGCTGGTATCACCCAGCCACGCCGACTGACCAAAGCTCAGACTGGTGACTTCGTTACTGGTCGTCCAGAAGACATCTCGTTCCTCCAACTGGAGAAGCAAGCAGACTTTACTGTAGCTAACGCCGTAAGTGACGCTATCGCGGCTCGCCTTTCGTTTGCCTTTATGTTGAACTCTGCGGTTCAGCGTACAGGTGAACGTGTGACCGCCGAAGAGATTCGGTATGTAGCTTCTGAACTTGAAGATACTTTAGGTGGTGTCTACTCTATACttt                                                                                                                                                    <  1:36221/301‑1 (MQ=255)`  
                                                                                                                                                        `gCTCTAAGGTTATCGGATTAGTGAATCCTGCTGGTATCACCCAGCCACGCCGACTGACCAAGGCTCAGACTGGTGACTTCGTTACTGGTCGTCCAGAAGACATCTCGTTCCTCCAACTGGAGAAGCAAGCAGACTTTACTGTAGCTAACGCCGTAAGTGACGCTATCGAGGCTCGCCTTTCGTTTGCCTTTATGTTGAACTCTGCGGTTCAGCGTACAGGTGAACGTGTGACCGCCGAAGGGATTCGGTATGTCGCTTCTGAACTTGAAGATACTTTAGGTGGTGTCTActcctgtctctt                                                                                                                                                   >  1:430074/1‑292 (MQ=255)`  
                                                                                                                                                               `ggTTATCGGCTTAGTGAATCCTGCTGGTATCACCCAGCCACGCCGACTGACCAAAGCTCAGACTGGTGACTTCGTTACTGGTCGTCCAGAAGACATCTCGTTCCTCCAACTGGAGAAGCAAGCAGACTTTACTGTAGCTAACGCCGTAAGTGACGCTATCGCGGCTCGCCTTTCGTTTGCCTTTATGTTGAACTCTGCGGTTCAGCGTACAGGTGAACGTGTGACCGCCGAAGAGATTCGGTATGTAGCTTCTGAACTTGAAGATACTTTAGGTGGTGTCTACTCTATCCTTTCTCAAGaa                                                                                                                                            >  1:317519/1‑301 (MQ=255)`  
                                                                                                                                                                          `tAGTGAATCCTGCTGGTATCACCCAGCCACGCCGACTGACCAAAGCTCAGACTGGTGACTTCGTTACTGGTCGTCCAGAAGACATCTCGTTCCTCCAACTGGAGAAGCAAGCAGACTTTACTGTAGCTAACGCCGTAAGTGACGCTATCGCGGCTCGCCTTTCGTTTGCCTTTATGTTGAACTCTGCGGTTCAGCGTACATGTGAACGTGTGACCGCCGAAGAGATTCGGTATGTAGCTTCTGAACTTGAAGATACTTTAGGTGGTGTCTACTCTATCCTTTCTCAAGAATTACAAtctgt                                                                                                                                 >  1:479834/1‑297 (MQ=255)`  
                                                                                                                                                                                 `agagacaggTATCACCCAGCCACGCCGACTGACCAAAGCTCAGACTGGTGACTTCGTTACTGGTCGTCCAGAAGACATCTCGTTCCTCCAACTGGAGAAGCAAGCAGACTTTACTGTAGCTAACGCCGTAAGTGACGCTATCGCGGCTCGCCTTTCGTTTGCCTTTATGTTGAACTCTGCGGTTCAGCGTACAGGTGAACGTGTGACCGCCGAAGAGATTCGGTATGTAGCTTCTGAACTTGAAGATACTTTAGGTGGTGTCTACTCTATCCTTTCTCAAGAATTACAATTGCCTCTGGTa                                                                                                                          <  1:732046/294‑1 (MQ=255)`  
                                                                                                                                                                                 `acaggctgGTATCACCCAGCCACGCCGACTGACCAAAGCTCAGACTGGTGACTTCGTTACTGGTCGTCCAGAAGACATCTCGTTCCTCCAACTGGAGAAGCAAGCAGACTTTACTGTAGCTAACGCCGTAAGTGCCGCTATCGAGGCTCGCCTTTCGTTTGCCTTTATGTTGAACTCTGCGGTTCAGCGTACAGGTGAACGTGTGACCGCCGAAGAGATTCGGTATGTAGCTTCTGAACTTGAAGATACTTTAGGTGGTGTCTACTCTATCCTTTCTCAAGAATTACAATTGCCTCTGGTa                                                                                                                          <  1:747458/297‑1 (MQ=255)`  
                                                                                                                                                                                   `ctgctgGTATCACCCAGCCACGCCGACTGACCAAAGCTCAGACTGGTGACTTCGTTACTGGTCGTCCAGAAGACATCTCGTTCCTCCAACTGGAGAAGCAAGCAGACTTTACTGTAGCTAACGCCGTAAGTGCCGCTATCGAGGCTCGCCTTTCGTTTGCCTTTATGTTGAACTCTGCGGTTCAGCGTACAGGTGAACGTGTGACCGCCGAAGAGATTCGGTATGTAGCTTCTGAACTTGAAGATACTTTAGGTGGTGTCTACTCTATCCCTTCTCAAGAATTACAATTGCCTCTGGTACg                                                                                                                        <  1:610170/301‑1 (MQ=255)`  
                                                                                                                                                                                     `gctgGTATCACCCAGCCACGCCGACTGACCAAAGCTCAGACTGGTGACTTCGTTACTGGTCGTCCAGAAGACATCTCGTTCCTCCAACTGGAGAAGCAAGCAGACTTTACTGTAGCTAACGCCGTAAGTGACGCTATCGCGGCTCGCCTTTCGTTTGCCTTTATGTTGAACTCTGCGGTTCAGCGTACAGGTGAACGTGTGACCGCCGAAGAGATTCGGTATGTAGCTTCTGAACTTGAAGATACTTTAGGTGGTGTCTAATCTATCCTTTCTTAAGAATTACAATTGCCTCTGGTACGAg                                                                                                                      >  1:549163/1‑301 (MQ=255)`  
                                                                                                                                                                                      `ctgGTTTCGCCCAGCCACGCCGCCTGACCAAAACTCAGACTGGTGACTTCGTTACTGGTCGTCCAGAAGACTGCTCGTTCCTCCAACTGGAGAAGCAAGCAGACTTTACGGTAGCTAACGCCGTAAGTGACGCTATCGCCGCTCGCCTTTCGTTTGCCTTTATGTTGAACTCTGCGGTTCAGCGTACAGGTGAACGTGTGACCGCCGAAGAGATTCGGTATGTAGCTTCTGAACTTGAAGATACTTTAGGTGGTGTCTACTCTATCCTTTCTCAAGAATTACAATTGCCTCTGGTACGAGt                                                                                                                     <  1:437234/301‑1 (MQ=255)`  
                                                                                                                                                                                        `ggTATCACCCCGCCACGCCGACTGACCAAAGCTCAGACTGGTGACTTCGTTACTGGTCGTCCAGAAGACATCTCGTTCCTCCAACTGGAGAAGCAAGCAGACTTTACTGTAGCTAACGCCGTAAGTGACGCTATCGCGGCTCGCCTTTCGTTTGCCTTTATGTTGAACTCTGCGGTTCAGCGTACAGGTGAACGTGTGACCGCCGAAGAGATTCGGTATGTAGCTTCTGAACTTGAAGATACTTTAGGTGGTGTCTACTCTATCCTTTCTCAAGAATTACAATTGCCTCTGGTACGAGTGc                                                                                                                   <  1:65733/301‑1 (MQ=255)`  
                                                                                                                                                                                        `ggTATCACCCAGCCACGCCGACTGACCAAAGCTCAGACTGGTGACTTCGTTACTGGTCGTCCAGAAGACATCTCGTTCCTCCAACTGGAGAAGCAAGCAGACTTTACTGTAGCTAACGCCGTAAGTGACGCTATCGCGGCTCGCCTTTCGTTTGCCTTTATGTTGAACTCTTCGGTTCAGCGTACAGGTGAACGTGTGACCGCCGAAGAGATTCGGTATGTAGCTTCTGAACTTGAAGATACTTTAGGTGGTGTCTACTCTATCCTTTCTCAAGAATTACAATTTCCTCTGGTACGAGTGc                                                                                                                   >  1:681749/1‑301 (MQ=255)`  
                                                                                                                                                                                        `ggTATCACCCAGCCACGCCGACTGACCAAAGCTCAGACTGGTGACTTCGTTACTGGTCGTCCAGAAGACATCTCGTTCCTCCAACTGGAGAAGCAAGCAGACTTTACTGTAGCTAACGCCGTAAGTGACGCTATCGCGGCTCGCCTTTCGTTTGCCTTTATGTTGAACTCTGCGGTTCAGCGTACAGGTGAACGTGTGACCGCCGAAGAGCTTCGGTATGTAGCTTCTGAACTTGAAGATACTTTAGGAGGTGTCTACTCTATCCTTTCTCAAGAATTACAATTGCCTCTGGTACGACTGc                                                                                                                   >  1:61760/1‑301 (MQ=255)`  
                                                                                                                                                                                              `aCCCAGCCACGCCGACTGACCAAAGCTCAGACTGGTGACTTCGTTACTGGTCGTCCAGAAGACATCTCGTTCCTCCAACTGGAGAAGCAAGCAGACTTTACTGTAGCTAACGCCGTAAGTGACGCTATCGCGGCTCGCCTTTCGTTTGCCTTTATGTTGAACTCTGCGGTTCAGCGTACAGGTGAACGTGTGACCGCCGAAGAGATTCGGTATGTAGCTTCTGAACTTGAAGATACTTTAGGTGGTGTCTACTCTATCCTTTCTCAAGAATTACAATTGCCTCTGGTACGAGTGCTCTTGa                                                                                                             >  1:407203/1‑301 (MQ=255)`  
                                                                                                                                                                                                 `cAGCCACGCCCACTGACCAAAGCCCAGACTGGTGACTTCGTTACTGGTCGTCCAGCAGACATCTCGTTCCTCCAACTGGAGAAGCAAGCAGACTTTACTGTAGCTAACGCCGTAAGTGACGCTATCGCGGCTCGCCTTTCGTTTGCCTTTATGTTGAACTCTGCGGTTCAGCGTACATGTGAACGTGTGACCGCCGAAGAGATTCGGTATGTAGCTTCTGAACTTGAAGATACTTTAGGTGGTGTCTACTCTATCCTTTCTCAAGAATTACAATTGCCTCTGGTACGAGTGCTCTTGAAGc                                                                                                          <  1:562851/301‑1 (MQ=255)`  
                                                                                                                                                                                                           `gACTGACCAAAGCTCAGACTGGTGACTTCGTTACTGGTCGTCCAGAAGACATCTCGTTCCTCCAGCTGGAGAAGCAAGCAGACTTTACTGTAGCTAACGCCGTAAGTTCCGCTATCGAGGCTCGCCTTTCGTTTGTCTTTATGTTGAACTCTGCGGTTCAGCGTACAGGTGAACGTGTGACCGCCGAAGAGATTCGGTATGTAGCTTCTGAACTTGAAGATACTTTAGGTGGTGTCTACTCTATCCTTTCTCAAGAATTACACTTGCCTCTGGTACGAGTGCTCTTgctgtctcttataca                                                                                                >  1:215234/1‑287 (MQ=255)`  
                                                                                                                                                                                                           `gACAGACCAAAGCCCAGACTGGTGACTTCGTTACTGGTCGTCCAGAAGTCATCTCGTTCCCCCAACTGGAGAAGAAAGCAGACTTTACTGTAGCTAACGCCGTAAGTGACGGTATCGAGGCTCGCCTTGCTAGTGCGTTTATGTTGAACTCTGAGGTTCAGCGTACAGGTGAACGTGTGACCGCCGAAGAGATTCGGTATGTAGCTTCTGAAATTGAAGATACTTTAGGTGGTGTCTACGCTATCCTTGCTCAAGAATTACAATTGCATCTGGTACGAGTGCTCTTGAAGCAACTACAAGc                                                                                                <  1:724183/301‑1 (MQ=255)`  
                                                                                                                                                                                                               `gACCAAAGCTCAGACTGGTGACTTCGTTACTGGTCGTCCAGAAGACATCTCGTCCCTCCAACTGGAGAAGCAAGCAGACTTTACTGTAGCTAACGCCGTAAGTGACGCTATCGCGGCTCGCCTTTCGTTTGCCTTTATGTTGAACTCTGCGGTTCAGCGTACAGGTGAACGTGTGACCGCCGAAGAGATTCGGTATGTAGCTTCTGAACTTGAAGATACTTTAGGTGGTGTCTACTCTATCCTTTCTCAAGAATTACAATTGCCTCTGGTACGAGTGCTCTTGAAGCAACTACAAGCCAcc                                                                                            >  1:69725/1‑300 (MQ=255)`  
                                                                                                                                                                                                                   `aaaGCTCAGACTGGTGACTTCGTTACTGGTCGTCCAGAAGACATCTCGTTCCTCCAACTGGAGAAGCAAGCAGACTTTACTGTAGCTAACGCCGTAAGTGACGCTATCGCGGCTCGCCTTTCGTTTGCCTTTATGTTGAACTCTGCGGTTCAGCGTACAGGTGAACGTGTGACCGCCGAAGAGATTCGGTATGTAGCTTCTGAACTTGAAGATACTTTAGGTGGTGTCTACTCTATCCTTTCTCAAGAATTACAATTGCCTCTGGTACGAGTGCTCTTGAAGCAACTACAAGCCACGCAAc                                                                                        <  1:753701/301‑1 (MQ=255)`  
                                                                                                                                                                                                                      `gCTCAGACTGGTGACTTCGTTACTGGTCGTCCAGAAGACATCTCGTTCCTCCAACTGGAGAAGCAAGCAGACTTTACTGTAGCTAACGCCGTAAGTGACGCTATCGCGGCTCGCCTTTCGTTTGCCTTTATGTTGAACTCTGCGGTTCAGCGTACAGGTGAACGTGTGACCGCCGAAGAGATTCGGTATGTAGCTTCTGAACTTGAAGATACTTTAGGTGGTGTCTACTCTATCCTTTCTCAAGAATTACAATTGCCTCTGGTACGAGTGCTCTTGAAGCAACTACAAGCCACGCAACAGa                                                                                     >  1:400299/1‑301 (MQ=255)`  
                                                                                                                                                                                                                          `aGACTGGTGACTTCGTTACTGGTCGTCCAGAAGACATCTCGTTCCTCCAACTGGAGAAGCAAGCAGACTTTACTGTAGCTAACGCCGTAAGTGCCGCTATCGAGGCTCGCCTTTCGTTTGCCTTTATGTTGAACTCTGCGGTTCAGCGTACAGGTGAACGTGTGACCGCCGAAGAGATTCGGTATGTAGCTTCTGAACTTGAAGATACTTTAGGTGGTGTCTACTCTATCCTTTCTCAAGAATTACAATTGCCTCTGGTACGAGTGCTCTTGAAGCAACTACAAGCCACGCAACAGATTcc                                                                                 >  1:817519/1‑301 (MQ=255)`  
                                                                                                                                                                                                                          `aGACTGGTGACTTCGTTACTGGTCGTCCAGAAGACATCTCGTTCCTCCAACTGGAGAAGCAAGCAGACTTTACTGTAGCTAACGCCGTAAGTGACGCTATCGCGGCTCGCCTTTCGTTTGCCTTTATGTTGAACTCTGCGGTTCAGCGTACAGGTGAACGTGTGACCGCCGAAGAGATTCGGTATGTAGCTTCTGAACTTGAAGATACTTTAGGTGGTGTCTACTCTATCCTTTCTCAAGAATTACAATTGCCTCTGGTACGAGTGCTCTTGAAGCAACTACAAGCCACGCAACAGATTcc                                                                                 <  1:303409/301‑1 (MQ=255)`  
                                                                                                                                                                                                                            `aCTGGTGACTTCGTTACTGGTCGTCCAGAAGACATCTCGTTCCTCCAACTGGAGAAGCAAGCAGACTTTACTGTAGCTAACGCCGTAAGTGACGCTATCGCGGCTCGCCTTTCGTTTGCCTTTATGTTGAACTCTGCGGTTCAGCGTACAGGTGAACGTGTGACCGCCGAAGAGATTCGGTATGTAGCTTCTGAACTTGAAGATACTTTAGGTGGTGTCTACTCTATCCTTTCTCAAGAATTACAATTGCCTCTGGTACGAGTGCTCTTGAAGCAACTACAAGCCACGCAACAGATTCCTg                                                                               <  1:511366/301‑1 (MQ=255)`  
                                                                                                                                                                                                                              `tGGTGACTTCGTTACTGGTCGTCCAGAAGACATCTCGTTCCTCCAACTGGAGAAGCAAGCAGACTTTACTGTAGCTAACGCCGTAAGTGACGCTATCGCGGCTCGCCTTTCGTTTGCCTTTATGTTGAACTCTGCGGTTCAGCGTACAGGTGAACGTGTGACCGACGAAGAGATTCGGTATGTAGCTTCTGAACTTGAAGATACTTTAGGTGGTGTCTACTCTATCCTTTCTCAAGAATTACAATTGCCTCTGGTACGAGTGCTCTTGAAGCAACTACAAGACACGCAACAGATTCCTGAg                                                                             <  1:638888/301‑1 (MQ=255)`  
                                                                                                                                                                                                                               `ggTGACTTCGTTACTGGTCGTCCAGAAGACATCTCGTTCCTCCAACTGGAGAAGCAAGCAGACTTTACTGTAGCTAACGCCGTAAGTGACGCTATCGCGGCTTGCCTTTCGTTTGCCTTTATGTTGAACTCTGCGGTTCAGCGTACAGGTGAACGTGTGACCGCCGAAGAGATTCGGTATGTAGCTTCTGAACTTGAAGATACTTTAGGTGGTGTCTACTCTATCCTTTCTCAAGAATTACAATTGCCTCTGGTACGAGTGCTCTTGAAGCAACTACAAGCCACGCAACAGATTCCTGAGt                                                                            <  1:769172/301‑1 (MQ=255)`  
                                                                                                                                                                                                                               `ggTGACTTCGTTACTGGTCGTCCAGAAGACATCTCGTTCCTCCAACTGGAGAAGCAAGCAGACTTTACTGTAGCTAACGCCGTAAGTGACGCTATCGCGGCTCGCCTTTCGTTTGCCTTTATGTTGAACTCTGCGGTTCAGCGTACAGGTGAACGTGTGACCGCCGAAGAGATTCGGTATGTAGCTTCTGAACTTGAAGATACTTTAGGTGGTGTCTACTCTATCCTTTCTCAAGAATTACAATTGCCTCTGGTACGAGTGCTCTTTAAGCAACTACAAGCCACGCACCAAATTCCTTAGt                                                                            >  1:19936/1‑301 (MQ=255)`  
                                                                                                                                                                                                                                         `ttACTGGTCGTCCAGAAGTCATCTCTTTCCTCCAACTGGAGAAGCAATCAGTCTTTACTGTAGCTAACGCCGTAAGTGCCGCTATCGCGGCTCGCCTTTCGTTTGCCTTTATGTTGAACTCTGCTGTTCATCGTACAGGTGAACGTGTGACCGCCGAAGTGATTCGGTATGTATCTTCTGAACTTGAAGATACTTTAGGTGGTGTCTACTCTATTCTTTCTCAAGAATTACAATTGCCTCTGGTACGAGTGCTCTTGAAGCAACTACAAGCCACGCAACAGATTCCTGAGTTACCTAAGGa                                                                  <  1:92785/301‑1 (MQ=255)`  
                                                                                                                                                                                                                                         `ttACTGGTCGTCCAGAAGACATCTCGTTCCTCCAACTGGAGAAGCAAGCAGACTTTACTGTAGCTAACGCCGTAAGTGACGCTATCGCGGCTCGCCTTTCGTTTGCCTTTATGTTGAATTCTGCGGTTCAGCGTACAGGTGAACGTGTGACCGCCGAAGAGATTCGGTATGTAGCTTCTGAACTTGAAGATACTTTAGGTGGTGTCTACTCTATCCTTTCTCAAGAATTACAATTGCCTCTGGTACGAGTGCTCTTGAAGCAACTACAAGCCACGCAACAGATTCCTGAGTTACCTAAGGa                                                                  <  1:689435/301‑1 (MQ=255)`  
                                                                                                                                                                                                                                         `ttACTGGTCGTCCAGAAGACATCTCGTTCCTCCAACTGGAGAAGCAAGCAGACTTTACTGTAGCTAACGCCGTAAGTGACGCTATCGCGGCTCGCCTTTCGTTTGCCTTTATGTTGAACTCTGCGGTTCAGCGTACAGGTGATCGTGTGACCGCCGAAGAGATTCGGTATGTAGCTTCTGAACTTGAAGATACTTTAGGTGGTGTCTACTCTATCCTTTCTCAAGAATTACAATTGCCTCTGGTACGAGTGCTCTTGGAGCAACTACAAGCCACGcctgtctcttatacacatctccgagc                                                                  >  1:444616/1‑276 (MQ=255)`  
                                                                                                                                                                                                                                         `ttACTGGTCGTCCAGAAGACATCTCGTTCCTCCAACTGGAGAAGCAAGCAGACTTTACTGTAGCTAACGCCGTAAGTGACGCTATCGCGGCTCGCCTTTCGTTTGCCTTTATGTTGAACTCTGCGGTTCAGCGTACAGGTGAACGTGTGACCGCCGAAGAGATTCGGTATGTAGCTTCTGAACTTGAAGATACTTTAGGTGGTGTCTACTCTATCCTTTCTCAAGAATTACAATTGCCTCTGGTACGAGTGCTCTTGAAGCAACTACAAGCCACGCAACAGATTCCTGAGTTACCTAAGGa                                                                  <  1:525604/301‑1 (MQ=255)`  
                                                                                                                                                                                                                                                    `ccAGAAGACATCTCGTTCCTCCAACTGGAGAAGCAAGCAGACGTTACTGTAGCTAACGCCGTAAGTGACGCTATCGCGGCTCGCCTTTCGTTTGCCTTTATGTTGAACTCTGCGGTTCAGCGTACAGGTGAACGTGTGACCGCCGAAGAGATTCGGTATGTAGCTTCTGAACTTGAAGATACTTTAGGTGGTGTCTACTCTATCCTTTCTCAAGAATTACAATTGCCTCTGGTACGAGTGCTCTTGAAGCAACTACAAGCCACGCAACAGATTCCTTAGTTACCTAAGGAAGCCGTAGAGc                                                       <  1:533713/301‑1 (MQ=255)`  
                                                                                                                                                                                                                                                     `cAGAAGACATCTCGTTCCTCCAACTGGAGAAGCAAGCAGACTTTACTGTAGCTAACGCCGTAAGTGACGCTATCGCGGCTCGCCTGTCGTTTGCCTTTATGTTGAACTCTGCGGTTCAGCGTACAGGTGAACGTGTGACCGCCGAAGAGATTCGGTATGTAGCTTCTGAACTGGAAGATACTTTAGGTGGTGTCTACTCTATCCTTTCTCAAGAATTACAATTGCCTCTGGTACGAGTGCTCTTGAAGCAACTACAGGCCACGCAACAGATTCCTGAGTTACCTAAGGAAGCCGTAGAGcc                                                      <  1:518161/301‑1 (MQ=255)`  
                                                                                                                                                                                                                                                       `gaagaCATCTCGTTCCTCCAACTGGAGAAGCAAGCAGACTTTACTGTAGCTAACGCCGTAAGTGACGCTATCGCGGCTCGCCTTTCGTTTGCCTTTATGTTGAACTCTGCGGTTCAGCGTACAGGTGAACGTGTGACCGCCGAAGAGATTCGGTATGTAGCTTCTGAACTTGAAGATACTTTAGGTGGTGTCTACTCTATCCTTTCTCAAGAATTACAATTGCCTCTGGTACGAGTGCTCTTGAAGCAACTACAAGCCACGCAACAGATTCCTGAGTTACCTAAGGAAGCCGTAGAGCCaa                                                    >  1:273753/1‑301 (MQ=255)`  
                                                                                                                                                                                                                                                                 `tgtgtataagagacaggaAGCAAGCAGACTTTACTGTAGCTAACGCCGTAAGTGACGCTATCGCGGCTCGCCTTTCGTTTGCCTTTATGTTGAACTCTGCGGTTCAGCGTACAGGTGAACGTGTGACCGCCGAAGAGATTCGGTATGTAGCTTCTGAACTTGAAGATACTTTAGGTGGTGTCTACTCTATCCTTTCTCAAGAATTACAATTGCCTCTGGTACGAGTGCTCTTGAAGCAACTACAAGCCACGCAACAGATTCCTGAGTTACCTAAGGAAGCCGTAGAGCCAACCATTAGTAc                                          <  1:782466/285‑1 (MQ=255)`  
                                                                                                                                                                                                                                                                 `ggctcggagatgtgtataagagacaggACTTTACTGTAGCTAACGCCGTAAGTGACGCTATCGCGGCTCGCCTTTCGTTTGCCTTTATGTTGAACTCTGCGGTTCAGCGTACAGGTGAACGTGTTACCGCCGAAGAGATTCGGTATGTAGCTTCTGAACTTGAAGATACTTTAGGTGGTGTCTACTCTATCCTTTCTCAAGAATTACAATTGCCTCTGGTACGAGTGCTCTTGAAGCAACTACAAGCCACGCAACAGATTCCTGAGTTACCTAAGGAAGCCGTAGAGCCAACCATTAGTAc                                          <  1:280664/275‑1 (MQ=255)`  
                                                                                                                                                                                                                                                                 `cGTTCCTCCAACTGGAGAAGCAAGCAGACTTTACTGTAGCTAACGCCGTAAGTGCCGCTATCGAGGCTCGCCTTTCGTTTGCCTTTATGTTGAACTCTGCGGTTCAGCGTACAGGTGAACGTGTGACCGCCGAAGAGATTCGGTATGTAGCTTCTGAACTTGAAGATACTTTAGGTGGTGTCTACTCTATCCTTTCTCAAGAATTACAATTGCCTCTGGTACGAGTGCTCTTGAAGCAACTACAAGCCACGCAACAGATTCCTGAGTTACCTAAGGAAGCCGTAGAGCCAACCATTAGTAc                                          <  1:496476/301‑1 (MQ=255)`  
                                                                                                                                                                                                                                                                    `tcggagatgtgtataagagacaggACTTTACTGTAGCTAACGCCGTAAGTGACGCTATCGCGGCTCGCCTTTCGTTTGCCTTTATGTTGAACTCTGCGGTTCAGCGTACAGGTGAACGTGTGACCGCCGAAGAGATTCGGTATGTAGCTTCTGAACTTGAAGATACTTTAGGTGGTGTCTACTCTATCCTTTCTCAAGAATTACAATTGCCTCTGGTACGAGTGCTCTTGAAGCAACTACAAGCCACGCAACAGATTCCTGAGTTACCTAAGGAAGCCGTAGAGCCAACCATGAGTACAgg                                       <  1:224337/278‑1 (MQ=255)`  
                                                                                                                                                                                                                                                                     `cctccAACTGGAGAAGCAAGCAGACTTTACTGTAGCTAACGCCGTAAGTGACGCTATCGCGGCTCGCCTTTCGTTTGCCTTTATGTTGAACTCTGCGGTTCAGCGTACAGGTGAACGTGTGACTGCCGAAGAGATTCGGTATGTAGCTTCTGAACTTGAAGATACTTTAGGTGGTGTCTACTCTATCCTTTCTCAAGAATTACAATTGCCTCTGGTACGAGTGCTCTTGAAGCAACTACAAGCCACGCAACAGATTCCTTAGTTACCTAAGGAAGCCGTAGAGCCAACCATTAGTACAGGt                                      >  1:240440/1‑301 (MQ=255)`  
                                                                                                                                                                                                                                                                            `cTGGAGAAGCAAGCAGACTTTACTGTAGCTAACGCCGTAAGTGACGCTATCGCGGCTCGCCTTTCGTTTGCCTTTATGTTGAACTCTGCGGTTCAGCGTACAGGTGAACGTGTGACCGCCGAAGAGATTCGGTATGTAGCTTCTGAACTTGAAGATACTTTAGGTGGTGTCTACTCTATCCTTTCTCAAGAATTACAATTGCCTCTGGTACGAGTGCTCTTGAAGCAACTACAAGCCACGCAACAGATTCCTAAGTTACCTAAGGAAGCCGTAGAGCCAACCATTAGTACAGGTCTGGAAg                               >  1:740264/1‑301 (MQ=255)`  
                                                                                                                                                                                                                                                                            `atGGAGAAGCAAGCAGACTTTACTGTAGCTAACGCCGTAAGTGACGCTATCGCGGCTCGCCTTTCGTTTGCCTTTATGTTGAACTCTGCGGTTCAGCGTACAGGTGAACGTGTGACCGCCGAAGAGATTCGGTATGTAGCTTCTGAACTTGAAGATACTTTAGGTGGTGTCTACTCTATCCTTTCTCAAGAATTACAATTGCCTCTGGTACGAGTGCTCTTGAAGCAACTACAAGCCACGCAACAGATTCCTGAGTTACCTAAGGAAGCCGTAGAGCCAACCATTAGTACAGGTCTGGAAg                               <  1:776540/300‑1 (MQ=255)`  
                                                                                                                                                                                                                                                                                 `gaAGCAAGCAGACTTTACTGTAGCTAACGCCGTAAGTGACGCTATCGCGGCTCGCCTTTCGTTTGCCTTTATGTTGAACTCTGCGGTTCAGCGTACAGGTGAACGTGTGACCGCCGAAGAGATTCGGTATGTAGCTTCTGAACTTGAAGATACTTTAGGTGGTGTCTACTCTATCCTTTCTCAAGAATTACAATTGCCTCTGGTACGAGTGCTCTTGAAGCAACTACAAGCCACGCAACAGATTCCTGAGTTACCTAAGGAAGCCGTAGAGCCAACCATTAGTACAGGTCTGGAAGCAAtt                          >  1:376264/1‑301 (MQ=255)`  
                                                                                                                                                                                                                                                                                          `aGACTTTACTGTAGCTAACGCCGTAAGTGACGCTATCGCGGCTCGCCTTTCGTTTGCCTTTATGTTGAACTCTGCGGTTCAGCGTACAGGTGAACGTGTGACCGCCGAAGAGATTCGGTATGTAGCTTCTGAACTTGAAGATACTTTAGGTGGTGTCTACTCTATCCTTTCTCAAGAATTACAATTGCCTCTGGTACGAGTGCTCTTGAAGCAACTACAAGCCACGCAACAGATTCCTGAGTTACCTAAGGAAGCCGTAGAGCCAACCATTAGTACAGGTCTGGAAGCAATTGGTCGAGGa                 >  1:665292/1‑301 (MQ=255)`  
                                                                                                                                                                                                                                                                                               `ttACTGTAGCTAACGCCGTAAGTGACGCTATCGCGGCTCGCCTTTCGTTTGCCTTTATGTTGAACTCTGCGGTTCAGCGTACAGGTGAACGTGTGACCGCCGAAGAGATTCGGTATGTAGCTTCTGAACTTGAAGATACTTTAGGTGGTGTCTACTCTATCCTTTCTCAAGAATTACAATTGCCTCTGGTACGAGTGCTCTTGAAGCAACTACAAGCCACGCAACAGATTCCTGAGTTACCTAAGGAAGCCGTAGAGCCAACCATTAGTACAGGTCTGGAAGCAATTGGTCGAGGACaaaa            >  1:185823/1‑299 (MQ=255)`  
                                                                                                                                                                                                                                                                                                `tACTGTAGCTGACGCCGTAAGTGACGCTATCGCGGCTCGCCTTTCGTTTGCCTTTATGTTGAACTCTGCGGTTCAGCGTACAGGTGAACGTGTGACCGCCGAAGAGATTCGGTATGTAGCTTCTGAACTTGAAGATACTTTAGGTGGTGTCTACTCTATCCTTTCTCAAGAATTACAATTGCCTCTGGTACGAGTGCTCTTGAAGCAACTACAAGCCACGCAACAGATTCCTGAGTTACCTAAGGAAGCCGTAGAGCCAACCATTAGTACAGGTCTGGAAGCAATTGGTCGAGGACAAGAc           <  1:127614/301‑1 (MQ=255)`  
                                                                                                                                                                                                                                                                                                `tACTGTAGCTAACGCCTTAAGTGACGCGATCTCGGCTCGCCTTTCGTTTGCCTTTATGTTGAACTCTGCGGTTCAGCGTACAGGTGAACGTGTGACCGCCGAAGATATTCGGTATGTAGCGTCTGAACTTGAAGATACTTTATGTGGTGTCTACTCTATCCTTTCTCAAGAATTACAATTGCCTCTGGTACGAGTGCTCTTGAAGCAACTACAAGCCACGCAACAGATTCCTGAGTTACCTAAGGAAGCCGTAGAGCCAACCATTAGGACAGGTATGGAAGCAATTGGTCGAGGACAAGAc           <  1:570687/301‑1 (MQ=255)`  
                                                                                                                                                                                                                                                                                                `tACTGTAGCTAACGCCGTAAGTGACGCTATCGCGGCTCGCCTTTCGTTTGCCTTTATGTTGAACTCTGCGGTTCAGCGTACAGGTGAACGTGTGACCGCCAAAGAGATTCGGTATGTAGCTTCTGAACTTGAAGATACTTTAGGTGGTGTCTACTCTATCCTTTCTCAAGAATTACAATTGCCTCTGGTACGAGTGCTCTTGAAGCAACTACAAGCCACGCAACAGATTCCTGAGTTACCTAAGGAAGCCGTAGAACCAACCATTAGTACAGGTCTGGAAGCAATTGGTCGAGGACAAGAc           <  1:711889/301‑1 (MQ=255)`  
                                                                                                                                                                                                                                                                                                      `aGCTAACTCCGTAAGTGCCGCTATCGAGGCTCGCCTTTCGTTTGCCTTTATGTTGAACTCTGCGGTTCAGCGTACAGGTGAACGTGTGACCGCCGAAGAGATTCGGTATGTAGCTTCTGAACTTGAAGATACTTTAGGTGGTGTCTACTCTATCCTTTCTCAAGAATTACAATTGCCTCTTGTACGAGTGCTCTTGAAGCAACTACAAGCCACGCAACAGATTCCTGAGTTACCTAAGGAAGCCGTAGAGCCAACCATTAGTACAGGTCTGGAAGCAATTGGTCGAGGACAAGACCtgtct     >  1:473480/1‑297 (MQ=255)`  
                                                                                                                                                                                                                                                                                                      `aGCTAACGCCGTAAGTGACGCTATCGCGGCTCGCCTTTCGTTTGCCTTTATGTTGAACTCTGCGGTTCAGCGTACAGGTGAACGTGTGACCGCCGAAGAGATTCGGTATGTAGCTTCTGAACTTGAAGATACTTTAGGTGGTGTCTACTCTATCCTTTCTCAAGAATTACAATTGCCTCTGGTACGAGTGCTCTTGAAGCAACTACAAGCCACGCAACAGATTCCTGAGTTACCTAAGGAAGCCGTAGAGCCAACCATTAGTACAGGTCTGGAAGCAATTGGTCGAGGACAAGACCTTGAt     <  1:415564/301‑1 (MQ=255)`  

                                                                                                                                                                                                                                                                                                            `|`                                                                                                                                                                                                                                                                                                           
`GACTTATCCTAAAGAGGCTTGCCCATACATCCCGATTCGGATGGTCAGACTAGATGGTGAATCCTACGGTCGTTCGTACATTGAGGAATACTTAGGTGACTTACGGTCCCTTGAAAATCTCCAAGAGGCTATCGTCAAGATGTCCATGATTAGCTCTAAGGTTATCGGCTTAGTGAATCCTGCTGGTATCACCCAGCCACGCCGACTGACCAAAGCTCAGACTGGTGACTTCGTTACTGGTCGTCCAGAAGACATCTCGTTCCTCCAACTGGAGAAGCAAGCAGACTTTACTGTAGCTAAAGCCGTAAGTGACGCTATCGAGGCTCGCCTTTCGTTTGCCTTTATGTTGAACTCTGCGGTTCAGCGTACAGGTGAACGTGTGACCGCCGAAGAGATTCGGTATGTAGCTTCTGAACTTGAAGATACTTTAGGTGGTGTCTACTCTATCCTTTCTCAAGAATTACAATTGCCTCTGGTACGAGTGCTCTTGAAGCAACTACAAGCCACGCAACAGATTCCTGAGTTACCTAAGGAAGCCGTAGAGCCAACCATTAGTACAGGTCTGGAAGCAATTGGTCGAGGACAAGACCTTGATAAG  >  NC_001604.1/20965‑21562`

  

Alignment Legend

Aligned base mismatch/match (shaded by quality score): `ATCG/ATCG` `< 3 ≤` `ATCG/ATCG` `< 11 ≤` `ATCG/ATCG` `< 25 ≤` `ATCG/ATCG` `< 36 ≤` `ATCG/ATCG` `< 38 ≤` `ATCG/ATCG`

Unaligned base: `atcg`    Masked matching base: `atcg`    Alignment gap: `‑`    Deleted base: `‑`
